# Case Report: Borderline Lepromatous Leprosy Therapy Complicated by Type 1 Leprosy Reaction and Adverse Reactions with Dapsone and Clofazimine

**DOI:** 10.4269/ajtmh.22-0637

**Published:** 2024-01-23

**Authors:** Takashi Matono, Shotaro Suzuki, Shuichi Mori, Manabu Ato

**Affiliations:** ^1^ Department of Infectious Diseases, Aso Iizuka Hospital, Fukuoka, Japan;; ^2^ Leprosy Research Center, National Institute of Infectious Diseases, Tokyo, Japan

## Abstract

Leprosy is a global health issue, causing long-term functional morbidity and stigma. Rapid diagnosis and appropriate treatment are important; however, early diagnosis is often challenging, especially in nonendemic areas. Here, we report a case of borderline lepromatous leprosy accompanied by dapsone-induced (neutropenia, anemia, and methemoglobinemia) and clofazimine-induced (skin discoloration and ichthyosis) side effects and type 1 leprosy reactions during administration of the multidrug therapy. The patient completely recovered without developing any deformities or visual impairment. To ensure early diagnosis and a favorable outcome, clinicians should be aware of the diminished sensation of skin lesions as a key physical finding and manage the drug toxicities and leprosy reactions appropriately in patients on multidrug therapy.

## INTRODUCTION

Leprosy is a neglected tropical disease caused by the *Mycobacterium leprae* complex, which is an intracellular pathogen with an affinity for macrophages, histiocytes, and Schwann cells.^1^ Leprosy is a global health issue because it causes long-term morbidity such as neuropathy, disability, deformity, and discrimination and is endemic particularly in India, Brazil, and Indonesia.^2^^,^^3^ Early detection and appropriate management are important for global leprosy control. In Japan, two to five leprosy cases have been reported annually in the past 5 years, of which 88% were among immigrants from endemic areas.^4^ The skin lesions of leprosy mimic sarcoidosis, cutaneous lupus erythematosus, cutaneous leishmaniasis, fungal infection, and other mycobacterial infections, and its clinical presentation and histopathological findings are diverse. Furthermore, the *M. leprae* complex cannot be cultured in artificial media in clinical laboratories. Therefore, early diagnosis is often challenging, especially in nonendemic areas.^5^ Here, we report an imported case of borderline lepromatous leprosy in a patient who experienced multiple side effects and a leprosy reaction while on multidrug therapy (MDT).

## CASE PRESENTATION

**Figure 1.**
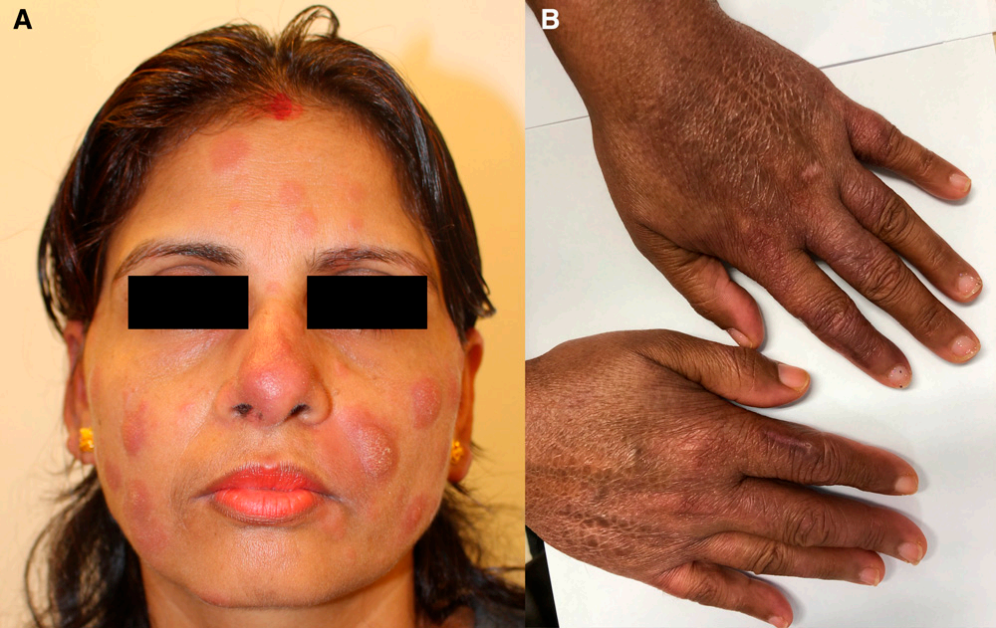
Skin lesions of borderline lepromatous leprosy and clofazimine-induced ichthyosis. (**A**) Multiple infiltrated reddish patches and plaques. (**B**) Clofazimine-induced ichthyosis and skin discoloration.

**Figure 2.**
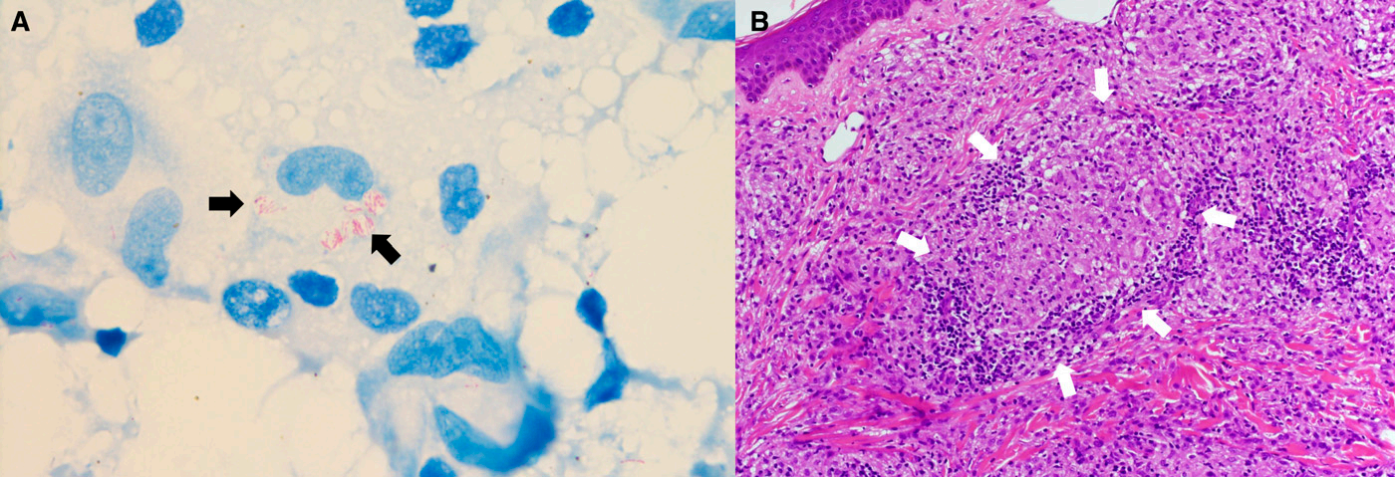
Histopathology of a skin biopsy. (**A**) Acid-fast bacilli in foamy macrophages (Ziehl–Neelsen staining, ×1,000). (**B**) An epithelioid granuloma in the dermis containing foamy macrophages, lymphocytes, and plasma cell infiltrates (hematoxylin and eosin staining, ×200).

**Figure 3.**
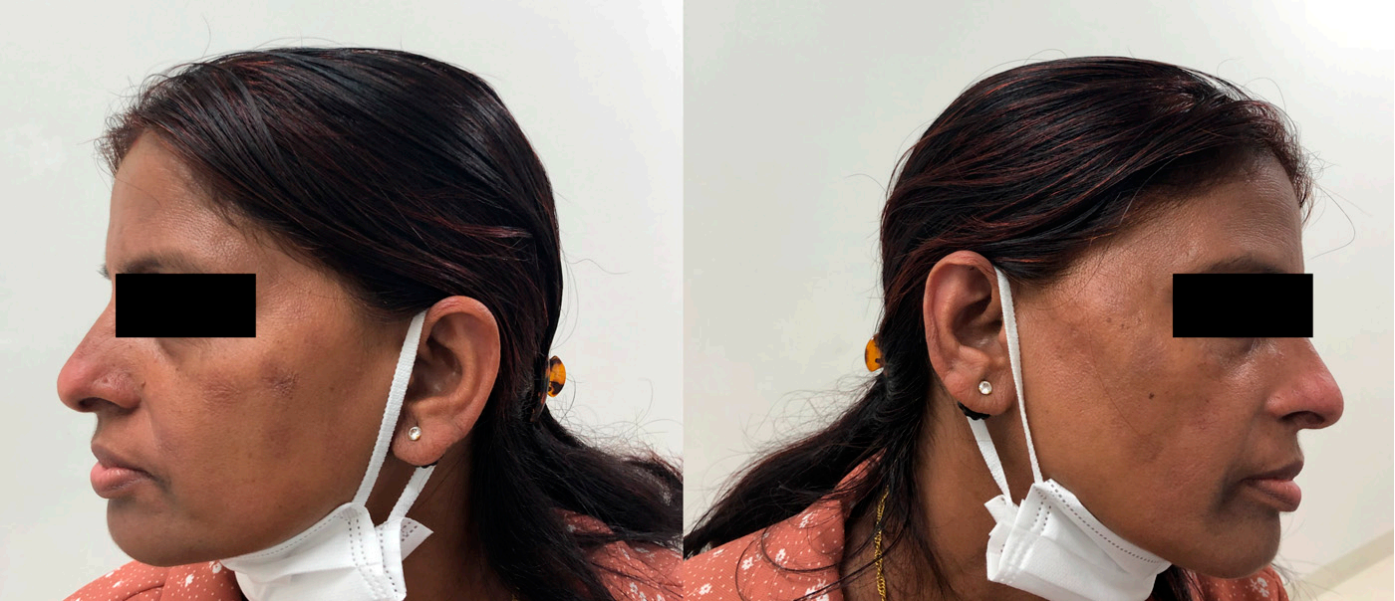
Cured leprosy skin lesions. Skin lesions at the time of multidrug therapy discontinuation.

A 36-year-old, previously healthy Nepalese woman, who had moved to Japan 3 years earlier, presented with a 1.5-year history of diffuse skin lesions. The lesions were generalized and involved the nose, face, trunk, legs, and arms. The patient had no history of close contact with armadillos or any person with leprosy. Physical examinations revealed multiple infiltrated reddish patches and plaques with partially pigmented desquamation (Figure 1) and diminished skin sensation on the lesions, but no peripheral nerve thickening or ophthalmic injuries. Ziehl–Neelsen staining of a skin biopsy specimen revealed acid-fast bacilli, and histopathology showed diffuse epithelioid granulomas in the dermis with foamy macrophages, lymphocytes, and plasma cell infiltrates (Figure 2). No mycobacteria were detected upon culturing of the specimen, and polymerase chain reaction (PCR) assays of the tissues for *Mycobacterium tuberculosis* complex and *Mycobacterium avium*-*intracellulare* complex were negative by COBAS TaqMan MTB/MAI (Roche Diagnostics, Tokyo, Japan). Based on the clinical diagnosis of borderline lepromatous leprosy (multibacillary forms; bacterial index [BI] = 4+), we administered dapsone at 100 mg daily, clofazimine at 300 mg once a month and 50 mg daily, and rifampicin at 600 mg once a month. All drugs were self-administered under direct observation by her husband with whom she lived. Although the serum phenolic glycolipid I (PGL-I) antibody test result (gelatin particle agglutination test) was negative, *M. leprae*–specific DNA (*M. leprae* RLEP and human β-globin) was detected by PCR of the skin biopsy specimen according to the method described in a previous report.^6^ One month after MDT initiation, the patient presented with headache and nausea. Physical examinations showed oxygen desaturation (oxygen saturation: 90% on room air). Hematology revealed neutropenia (neutrophils: 40/μL), anemia (hemoglobin: 8.1 g/dL), and methemoglobinemia (methemoglobin level: 12.9%). After discontinuing MDT for 10 days owing to the dapsone-induced side effects, we initiated a second-line regimen, including ofloxacin at 400 mg daily, clofazimine, and rifampicin because no mutations in drug resistance–determining regions in *rpoB* and *gyrA* were identified by PCR-direct sequencing using the skin biopsy specimen, according to the method published by the WHO.^7^ Three months after the MDT initiation, the patient’s preexisting skin lesions worsened, accompanied by edema and pain without neuritis. She was diagnosed with a mild type 1 leprosy reaction, and symptoms were managed using analgesics (nonsteroidal antiinflammatory drugs) for 3 months. She also experienced clofazimine-induced skin discoloration and ichthyosis (Figure 1). She eventually recovered after being on MDT for 3 years, with the disappearance of active lesions and a BI = 0 by skin biopsies according to the Japanese Leprosy Treatment Guidelines,^8^ without developing deformities or visual impairment (Figure 3). The final WHO disability gradings were grade 0 for the eyes and grade 1 for the hands and feet.^9^

## DISCUSSION

We describe a case of borderline lepromatous leprosy accompanied by dapsone- and clofazimine-induced side effects and a type 1 leprosy reaction. Herein, we discuss the diagnosis and treatment of leprosy.

First, diagnostic delay in leprosy is common and can lead to neurological damage or deformity. Since the introduction of free MDT in 1981, the reported number of new leprosy cases has significantly decreased globally, from 5.4 million in the mid-1980s to 127,558 in 2020.^3^^,^^10^ However, globally, 5.6% of patients have grade 2 disability at the time of diagnosis,^3^ reflecting the challenge of early diagnosis. In nonendemic areas, delay in diagnosis is common. In a study conducted in London, United Kingdom,^5^ 82% of patients had a delayed diagnosis, with a median time from symptom onset to diagnosis of 1.8 years; this is consistent with the experience of our patient who received a diagnosis 1.5 years after symptom onset. Diagnostic tests are not routinely available in clinical settings, and their sensitivity is relatively low. Specifically, the sensitivity of antibody (mostly targeting PGL-I antigen) and PCR tests is 64–72% and 75–89%, respectively.^11^ Therefore, leprosy is often diagnosed clinically, and decreased sensation of skin lesions is a key physical finding for making a clinical diagnosis. Furthermore, it is important to extract skin smear or skin biopsy specimens from the edge of skin lesions, and the sensitivity of Fite–Faraco staining (75–77%) is higher than that of Ziehl–Neelsen staining (57–59%).^12^^,^^13^ To facilitate early diagnosis of leprosy, clinicians should be aware of the endemic areas and typical clinical findings, even in nonendemic countries.

Second, treating leprosy with MDT is often challenging due to various side effects and leprosy reactions, as in this patient. The standard dose of rifampicin for leprosy is relatively nontoxic. However, dapsone, a sulfone antibiotic that inhibits folate synthesis, can cause serious side effects, including hypersensitivity reactions (drug rash with eosinophilia and systemic syndrome), methemoglobinemia, hemolytic anemia, neutropenia, agranulocytosis, and hepatotoxicity. Hemolytic anemia can occur in the early phase after MDT initiation, particularly in patients with glucose-6-phosphate dehydrogenase deficiency. As in this patient, methemoglobinemia causes tissue hypoxia. Therefore, prompt suspicion and evaluation of the side effects are important because the side effects are potentially life-threatening. Furthermore, clofazimine, a first-line drug, can frequently cause gastrointestinal symptoms and cosmetic defects such as hyperpigmentation and ichthyosis because it accumulates in adipose tissue and macrophages. Thus, healthcare professionals should inform patients of MDT-induced side effects and monitor patients on MDT to facilitate early detection of side effects. Compared with the traditional MDT regimens, a monthly regimen comprising rifampin, ofloxacin/moxifloxacin, and minocycline has recently been considered a less toxic and well-tolerated treatment option for not only paucibacillary leprosy but also multibacillary leprosy.^14^^–^^16^

Third, it is crucial to recognize that MDT can trigger type 1 and type 2 leprosy reactions, in addition to the side effects. The mechanism of type 1 and type 2 leprosy reactions involves a cellular immune response to a bacterial antigen and immune complex deposition in tissues and blood vessels, respectively.^2^ Type 1 leprosy reactions, as observed in our case, generally occur within 6 months after MDT initiation.^17^ In particular, borderline leprosy is considered a robust risk factor for type 1 leprosy reactions.^18^ A Nepalese study showed that 28% of patients with borderline leprosy developed type 1 reactions.^19^ These reactions require timely detection and effective treatment because they can cause nerve damage and impair quality of life, particularly in patients with nerve and ophthalmic symptoms. As in this patient, mild leprosy reactions can resolve by administering analgesics^8^^,^^20^; however, severe reactions may require systemic prednisone (against type 1 and 2 leprosy reactions) and thalidomide (against type 2 leprosy reactions).^21^ Recently, immunosuppressive drugs, including cyclosporine and methotrexate, have been studied as alternatives (steroid-sparing regimens) for severe type 1 leprosy reactions.^22^^–^^24^ Hence, for successful management and prevention of nerve damage, clinicians must anticipate the reactions in patients on MDT and address them promptly. In addition, it is crucial to manage disabilities and educate patients on self-care of the hands and feet to prevent secondary injuries, as in this patient with grade 1 disability.

In conclusion, early leprosy diagnosis is vital in both endemic and nonendemic areas to prevent long-term morbidity, including neuropathy, disability, and deformity. The present case illustrates the importance of clinicians being aware of the key clinical signs (e.g., diminished sensation of skin lesions) to ensure rapid diagnosis and appropriate management of drug side effects and leprosy reactions while administering MDT for a favorable outcome.
